# Inhibition of the Type III Secretion System of Salmonella enterica Serovar Typhimurium via Treatment with Fraxetin

**DOI:** 10.1128/spectrum.02949-22

**Published:** 2022-11-15

**Authors:** Yunjia Shi, Zeyu Sun, Yang Liu, Jingyan Shu, Yong Zhang, Qianghua Lv, Jianfeng Wang, Xuming Deng, Hongtao Liu, Jiazhang Qiu

**Affiliations:** a State Key Laboratory for Zoonotic Diseases, Key Laboratory for Zoonosis Research of the Ministry of Education, College of Veterinary Medicine, Jilin University, Changchun, China; b Department of Respiratory Medicine, Center for Pathogen Biology and Infectious Diseases, Key Laboratory of Organ Regeneration and Transplantation of the Ministry of Education, State Key Laboratory for Zoonotic Diseases, The First Hospital of Jilin University, Changchun, China; Texas A&M University

**Keywords:** fraxetin, *S.* Typhimurium, SPI-1, T3SS

## Abstract

The increasingly serious problem of bacterial drug resistance has led to the development of antivirulence agents. The Salmonella enterica serovar Typhimurium *Salmonella* pathogenicity island (SPI)-encoded type III secretion system (T3SS) and its effector proteins are important virulence factors for *S.* Typhimurium invasion and replication in host cells and for antivirulence drug screening. Fraxetin is isolated from *Fraxinus* spp. Extensive studies have reported its multiple pharmacological activities. However, it remains to be elucidated whether fraxetin affects the function of the *S.* Typhimurium T3SS. In this study, the anti-infection mechanism of fraxetin on *S.* Typhimurium and its T3SS was investigated. Fraxetin inhibited the *S.* Typhimurium invasion of HeLa cells without affecting the growth of bacteria *in vitro*. Further findings on the mechanism showed that fraxetin had an inhibitory effect on the *S.* Typhimurium T3SS by inhibiting the transcription of the pathogenesis-related SPI-1 transcriptional activator genes *hilD*, *hilC*, and *rtsA*. Animal experiments showed that fraxetin treatment protected mice against *S.* Typhimurium infection. Collectively, we provide the first demonstration that fraxetin may serve as an effective T3SS inhibitor for the development of treatments for *Salmonella* infection.

**IMPORTANCE** The increasingly serious problem of bacterial antibiotic resistance limits the clinical application of antibiotics, which increases the need for the development of antivirulence agents. The type III secretion system (T3SS) plays a critical role in host cell invasion and pathogenesis of *Salmonella* and becomes a popular target for antivirulence agents screening. Our study found, for the first time, that fraxetin inhibited *S.* Typhimurium invasion by inhibiting the transcription of genes in a feed-forward regulatory loop. Further *in vivo* testing showed that fraxetin decreased bacterial burdens in the spleen and liver of *S.* Typhimurium-infected mice and improved survival outcomes in an *in vivo* mouse model of *S.* Typhimurium infection. Collectively, these results demonstrate that fraxetin inhibits *S.* Typhimurium infection by targeting the T3SS and may serve as a potential agent for the treatment of *S.* Typhimurium infection.

## INTRODUCTION

Salmonella enterica serovar Typhimurium is a foodborne zoonotic and facultative intracellular enteric pathogen that is a common cause of intestinal infections in humans and animals worldwide (1). The emergence and spread of antibiotic-resistant bacteria is one of the greatest threats to human medicine, veterinary medicine, and public health (2). The emergence of multidrug-resistant *S.* Typhimurium is now a global public health emergency (3). The increasingly serious problem of bacterial antibiotic resistance limits the clinical application of antibiotics, which leads to the development of antivirulence agents (4).

*Salmonella* has developed a variety of virulence strategies to escape host immune defense and interact with the epithelium (5). *S.* Typhimurium delivers effector proteins into host cells via the type III secretion system (T3SS), facilitating its invasion and replication in host cells (6). The *Salmonella* pathogenicity island (SPI) is involved in nonphagocytic cell invasion and also induced intestinal inflammatory responses and diarrhea (7). The T3SS and its effector proteins encoded by SPI-1 genes are important virulence factors for *S.* Typhimurium invasion of host cells (8, 9) and consequently become targets for antivirulence drug screening (10, 11).

Fraxetin is the major extract component from an ash tree (*Fraxinus* spp.) (12). Fraxetin has diverse pharmacological properties, such as antimetastatic (13), antitumor (14), hepatoprotective (15), neuroprotective (16), antifibrotic (17), antihyperglycemic (18), and antioxidant activities (19, 20). Fraxetin inhibits Staphylococcus aureus proliferation by preventing topoisomerase from binding to DNA and blocking nucleic acid and protein synthesis (21). Even though fraxetin has many functions, its pharmacological activity against *S.* Typhimurium infection remains to be elucidated.

In this study, fraxetin was first demonstrated to be an antivirulence inhibitor of *S.* Typhimurium and a potential compound for treating *S.* Typhimurium infection. The discovery of the antivirulence function of fraxetin enriched its functional research and further expanded its clinical application.

## RESULTS

### Fraxetin inhibits the *S.* Typhimurium invasion of HeLa cells.

**FIG 1:**
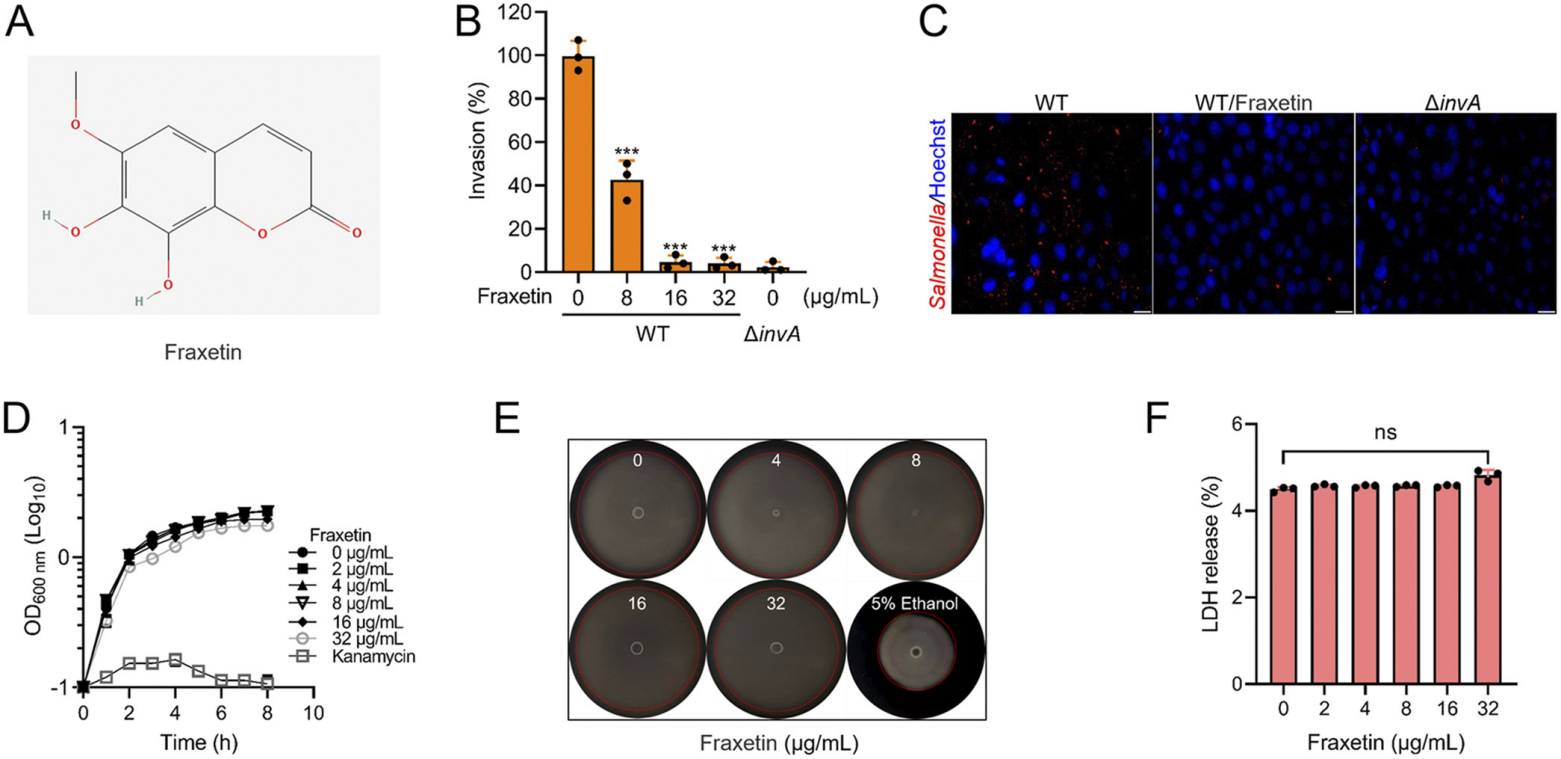
Fraxetin inhibits *S.* Typhimurium invasion of HeLa cells. (A) The chemical structure of fraxetin (CAS number 574-84-5; molecular formula is C_10_H_8_O_5_; molecular weight is 208.17). (B) Fraxetin inhibits *S.* Typhimurium invasion of HeLa cells. The inhibitory effect was determined by gentamicin protection assay. The invasion ratio of the control group (0 μg/mL fraxetin) was set as 100%. (C) Immunofluorescence results showed that fraxetin inhibited *S.* Typhimurium invasion of HeLa cells. Scale bar, 10 μm. (D) Less than or equal to 32 μg/mL of fraxetin has no effect on *S.* Typhimurium growth. Kanamycin (32 μg/mL) was used as a positive control. (E) Less than or equal to 32 μg/mL of fraxetin has no effect on *S.* Typhimurium swimming motility. Ethanol, applied at a final concentration of 5%, was used as a positive control. (F) Less than or equal to 32 μg/mL of fraxetin has no cytotoxicity on HeLa cells. The control group (0 μg/mL fraxetin) was added with the same volume of DMSO. NS, no significance; ***, *P < *0.001; bar, standard deviation (SD).

Single compounds isolated from traditional Chinese herbs are abundant sources of antivirulence agents. To identify T3SS inhibitors, we screened natural compounds using invasion assay of HeLa cells and found that 64 μg/mL of fraxetin inhibits the *S.* Typhimurium invasion of HeLa cells. Fraxetin (Fig. 1A) is a derivative of coumarin and is one of the main constituents of *Fraxinus* spp. The effect of fraxetin on *S.* Typhimurium infection has not been reported. The influence of fraxetin on *S.* Typhimurium invasion of HeLa cells was investigated first. The MIC of fraxetin against *S.* Typhimurium was higher than 1,024 μg/mL. The gentamicin protection assay was used to examine the effects of fraxetin on bacterial invasion into HeLa cells. Based on the results of the invasion assay, the proportion of bacteria invading cells in the wild-type (WT)-infected group was calculated as 100%, and the group treated with 16 μg/mL and 32 μg/mL fraxetin showed a 92% inhibitory rate (Fig. 1B). The rate of cell invasion with bacteria was similar to that of the Δ*invA* strain-infected group (Fig. 1B). The immunofluorescence results also suggested that fraxetin could inhibit the *S.* Typhimurium invasion of host cells compared with the WT-infected group (Fig. 1C). Flagella are necessary for bacterial attachment and interaction (22). To determine the reason for the inhibitory effect of fraxetin on invasion, the growth curve was measured in Luria-Bertani (LB) broth and the swimming mobility assay was conducted on semisolid motility agar. The results showed that fraxetin at less than or equal to 32 μg/mL had no effect on growth (Fig. 1D) or swimming mobility (Fig. 1E). Further lactate dehydrogenase (LDH) release assays showed that fraxetin had no cytotoxicity on HeLa cells (Fig. 1F). The results showed that fraxetin could decrease the *S.* Typhimurium invasion of host cells but had no effect on bacterial growth, swimming motility, or cytotoxicity in HeLa cells.

### Fraxetin restrains the translocation of SipA-TEM.

**FIG 2:**
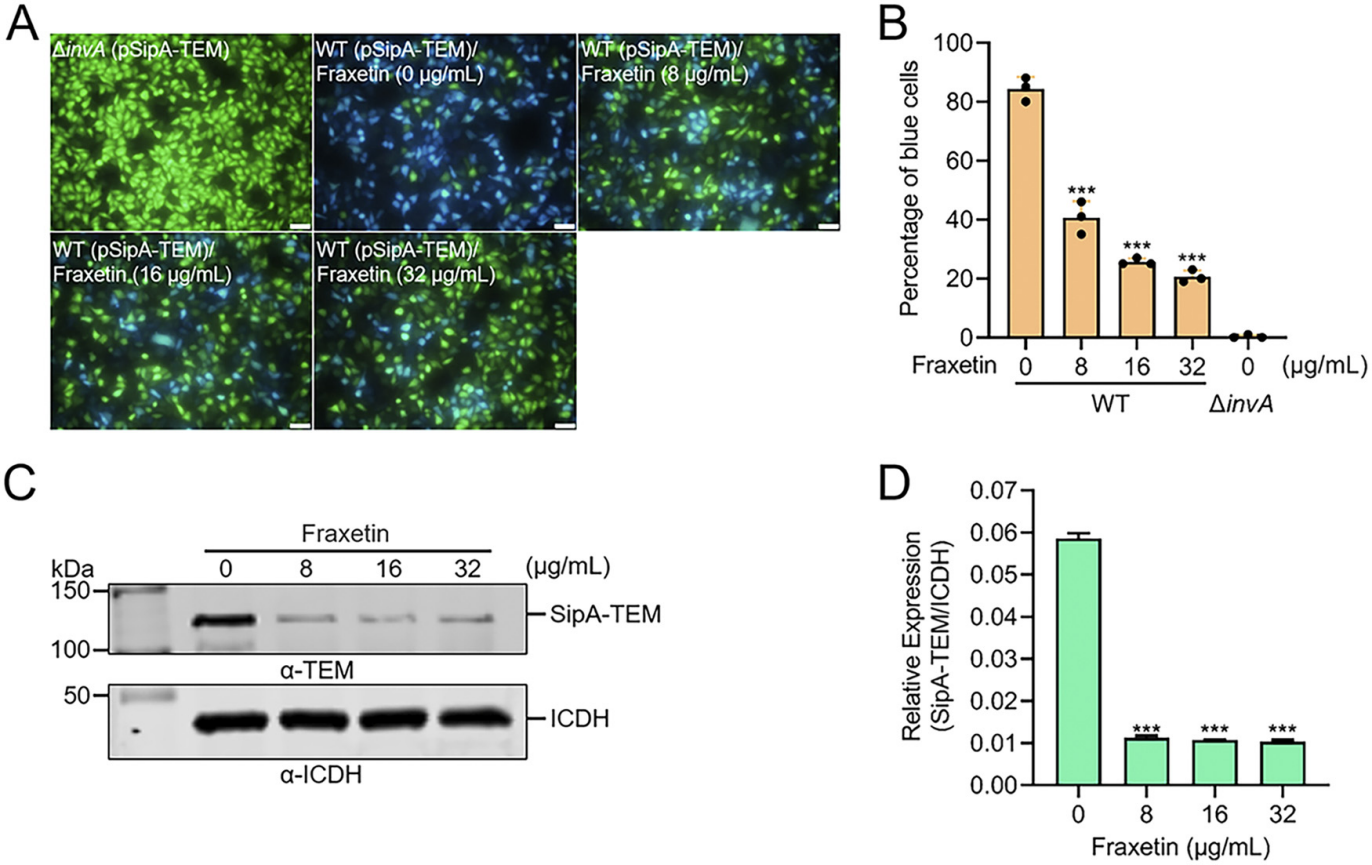
Fraxetin inhibits the translocation of SipA-TEM into HeLa cells. (A) The β-lactamase (TEM) assay showed that fraxetin inhibits the translocation of SipA-TEM into HeLa cells. Scale bar, 50 μm. (B) The inhibitory effect of fraxetin on SipA-TEM translocation into HeLa cells enhanced in a fraxetin dose-dependent manner. (C) Western blotting (WB) showed that fraxetin inhibited the expression of SipA-TEM. ICDH was used as a loading control. (D) Gray value of Western blotting was measured using ImageJ software. Each value represents an average of three biological replicates. The control group (0 μg/mL fraxetin) was added with the same volume of DMSO. ***, *P < *0.001; bar, SD.

To analyze the underlying inhibition mechanism of fraxetin in the invasion of HeLa cells, the translocation of SPI-1 genes encoding effector proteins, which are important for the invasion of host cells, was evaluated via TEM. HeLa cells were blue when CCF4 was added to the cells hydrolyzed by TEM, while unhydrolyzed CCF4 was green. Most HeLa cells (82%) infected with WT bacteria expressing the SipA-TEM fusion were blue due to hydrolyzation of CCF4/AM by SipA-TEM translocated into cells. The cells infected with Δ*invA* expressing SipA-TEM were green (100%) (Fig. 2A). The percentage of blue cells in the fraxetin-treated groups was higher than that in the WT-infected group (Fig. 2A and B). The proportions of blue cells in the 8 μg/mL, 16 μg/mL, and 32 μg/mL fraxetin-treated groups were 39%, 23%, and 19.5%, respectively (Fig. 2B). Western blotting (WB) results showed that fraxetin could effectively block the expression of SipA-TEM at a concentration of 8 μg/mL (Fig. 2C and D). These results indicate that fraxetin inhibits SipA-TEM expression and translocation into HeLa cells.

### Fraxetin decreases the secretion of T3SS effector proteins.

**FIG 3:**
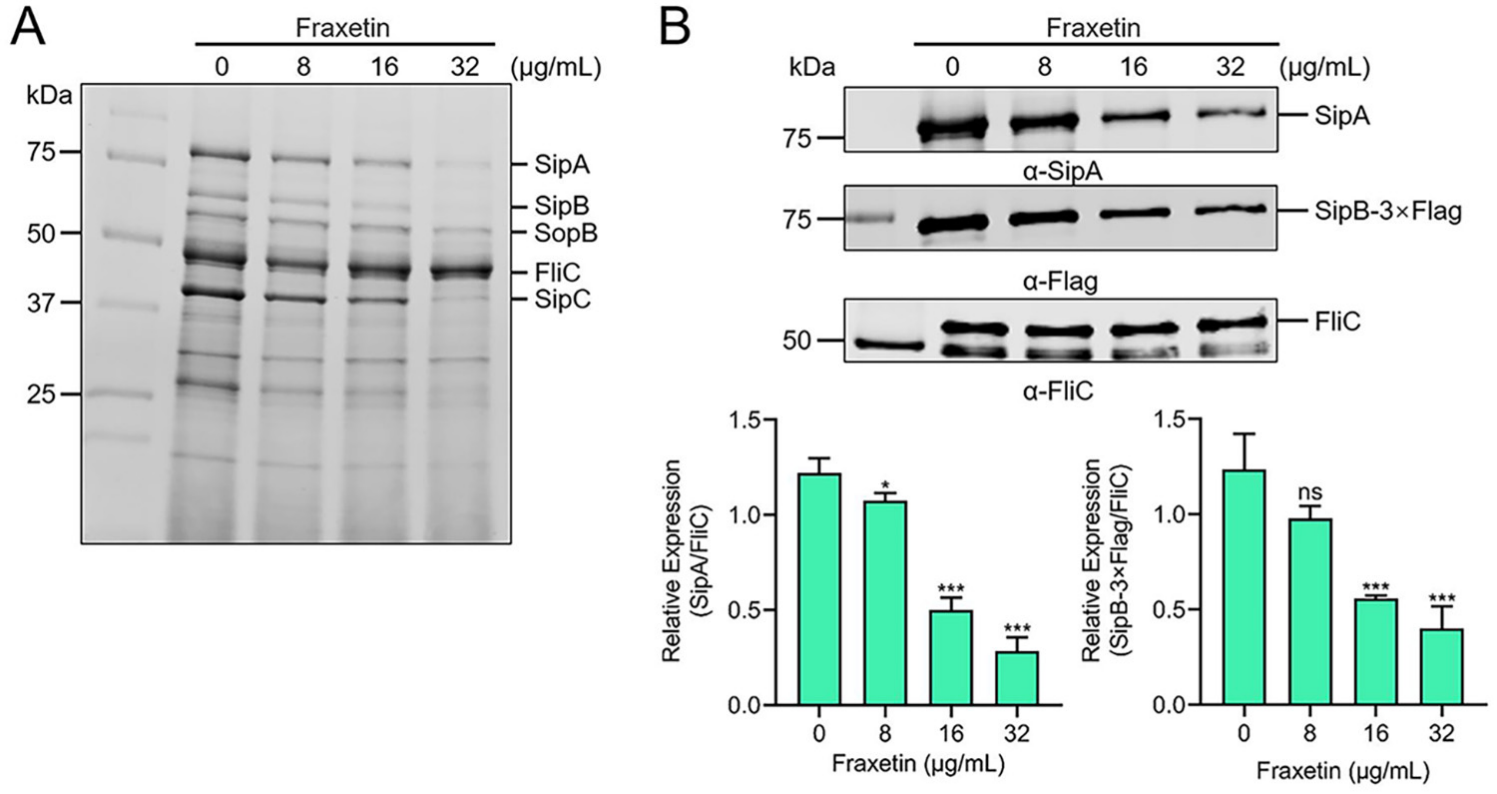
Fraxetin inhibits the secretion of T3SS effector proteins. (A) The SDS-PAGE analysis on inhibitory effect of fraxetin on the secretion of T3SS effector proteins. (B) The identity of the elution fractions was analyzed by WB. ImageJ software was used to quantify gray value of WB band. FliC was used as the loading control. Each value represents an average of three biological replicates. The control group (0 μg/mL fraxetin) was added with the same volume of DMSO. NS, no significance; *, *P < *0.05; ***, *P < *0.001; bar, SD.

Fraxetin effectively blocked the expression of SipA-TEM in SL1344 (Fig. 2). We wondered whether the expression of SipA and other SPI-1-encoded proteins was inhibited by fraxetin. To assess the impact of fraxetin on *S.* Typhimurium translocation, the bacterial culture supernatants coincubated with fraxetin were precipitated by trichloroacetic acid (TCA). The results of SDS-PAGE analysis showed that fraxetin at concentrations equal to or higher than 16 μg/mL inhibited the secretion of the SPI-1-encoded effector proteins SipA, SipB, SipC, and SopB (Fig. 3A). WB analysis further confirmed the inhibition by fraxetin (Fig. 3B). The inhibition was enhanced in a dose-dependent manner. Thus, fraxetin can block type III secretion.

### Fraxetin inhibits the expression of T3SS effector and regulatory genes.

**FIG 4:**
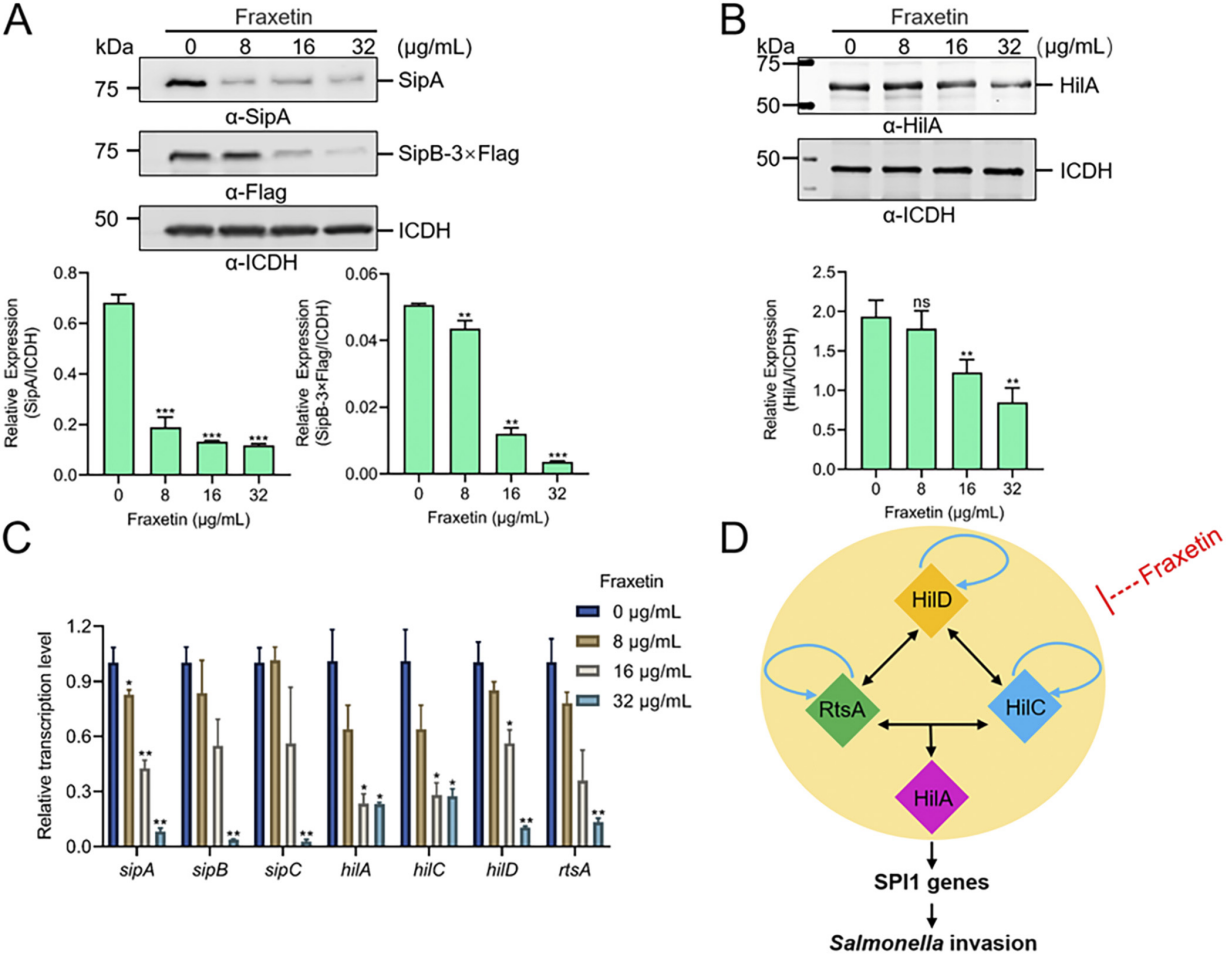
Fraxetin alleviates the transcription of T3SS effectors and regulatory genes. (A) WB analysis of T3SS effector expression of fraxetin-treated *S.* Typhimurium. (B) WB analysis of HilA expression in *S.* Typhimurium treated with fraxetin. ICDH was used as a loading control. (C) The qRT-PCR analysis of transcriptional level of T3SS effector and regulatory genes in *S.* Typhimurium treated with fraxetin. (D) A simplified model of the effect of fraxetin on feed-forward regulatory loop of T3SS. Black lines indicate transcriptional regulation. Blue lines indicate the proposed autoregulation of RtsA, HilD, and HilC. Repression is indicated as blunt lines and activation as arrows. The control group (0 μg/mL fraxetin) was added with the same volume of DMSO. NS, no significance; *, *P < *0.05; **, *P < *0.01; ***, *P < *0.001; bar, SD.

The above findings indicated the inhibition of fraxetin for T3SS secretion. To further investigate the mechanism of the inhibition, we analyzed the expression levels of the related effector proteins and regulatory genes by WB and quantitative real-time PCR (qRT-PCR). HilA is a transcriptional activator encoded on SPI-1 and controls the level of *S.* Typhimurium SPI-1 gene expression. The expression of T3SS effector proteins (SipA, SipB, and SipC) and the transcriptional activator HilA was measured by WB in samples treated with dimethyl sulfoxide (DMSO) or different concentrations of fraxetin. The lower expression level was observed in the fraxetin treatment groups compared to the DMSO control (Fig. 4A and B). These results demonstrate that fraxetin exhibits enhanced inhibitory activity on the expression of effector and regulatory genes in a concentration-dependent manner. The decreased expression level is most likely due to the decreased transcriptional level of genes. Therefore, the mRNA levels of effector and regulatory genes were measured by qRT-PCR. The results showed that the relative transcriptional levels of the effector genes *sipA*, *sipB*, and *sipC* in the fraxetin-treated group were lower than the levels in the untreated group (Fig. 4C). The inhibition rate of 32 μg/mL fraxetin was higher than 90% (Fig. 4C). The relative transcription of the regulatory genes *hilA*, *hilD*, *hilC*, and *rtsA* was also inhibited by fraxetin (Fig. 4C). The results of the above *in vitro* assay are summarized in Fig. 4D and showed that fraxetin affects the transcription and expression of the feed-forward regulatory loop of T3SS (Fig. 4D).

### Fraxetin effectively protects mice from *S.* Typhimurium infection.

**FIG 5:**
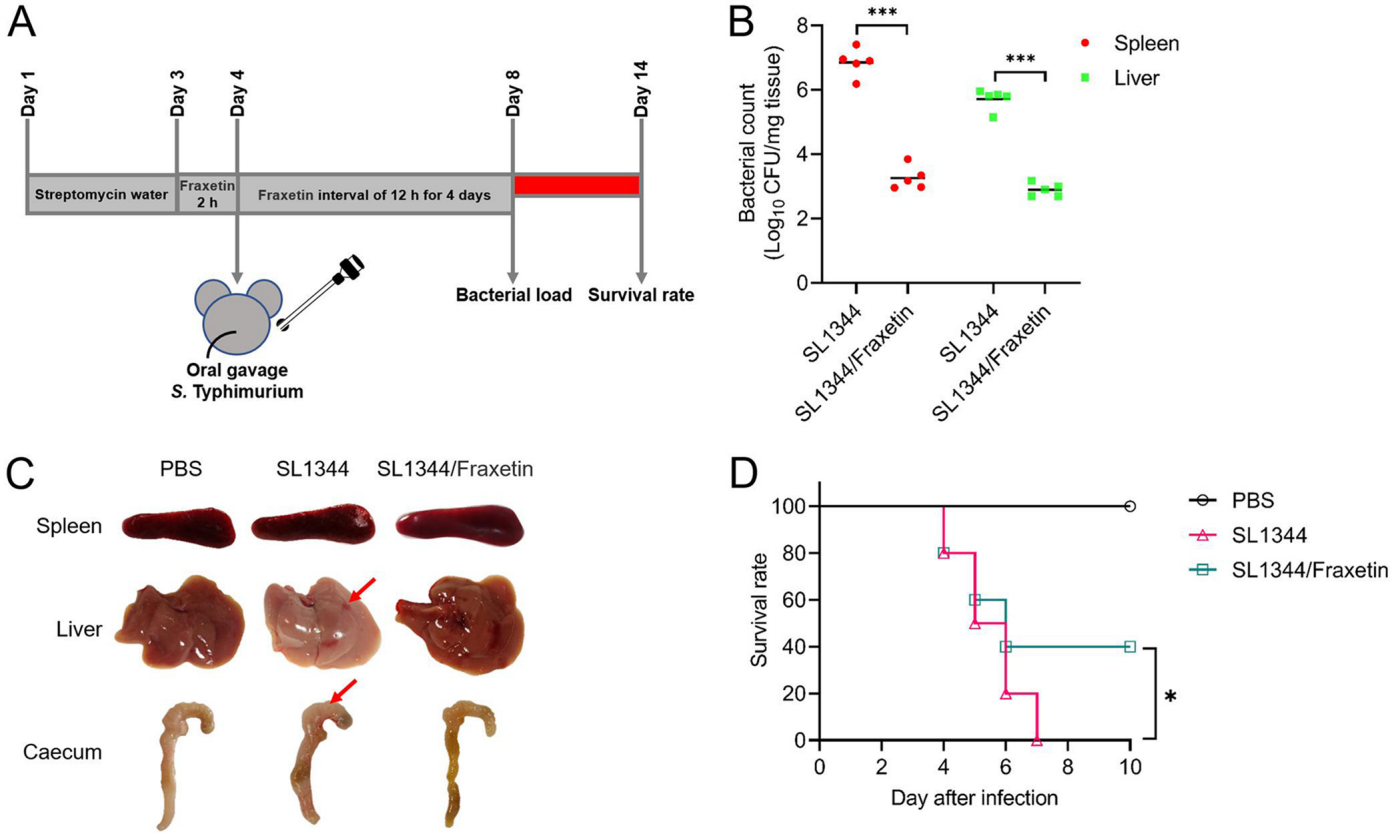
Fraxetin provides protection against *S.* Typhimurium infection in mice. (A) The workflow of the mouse infection. (B) Bacterial load in the livers and spleens of SL1344-infected mice 4 days postinfection. (C) The gross lesion observations of each group. (D) The survival rate was performed in GraphPad Prism 8.0. *, *P* < 0.05; ***, *P* < 0.001.

All of the *in vitro* results indicate that fraxetin has antivirulence activity against HeLa cell invasion by targeting the T3SS. To further analyze whether fraxetin has an anti-infection effect on SL1344-infected mice, each group of mice received streptomycin 72 h prior to infection with SL1344 (Fig. 5A). Mice were killed on the 4th day postinfection, and bacterial colonization was determined (Fig. 5A). The necropsy results combined with colony counts showed that the spleen and liver from fraxetin-treated mice had lower bacterial burdens than the control group (Fig. 5B). Examples of macroscopic observations are shown in Fig. 5C. The cecum contained less-solidified feces in the SL1344 group, whereas healthy and solidified feces were observed in the SL1344 + fraxetin group. The end of the cecum in the SL1344-infected group was smaller than that in the fraxetin-treated group mice. Hemorrhagic spots and gray on the liver surface were observed in the SL1344 group, whereas less destruction was observed in the fraxetin-treated group. According to these data, fraxetin decreases the colonization of target organs. The survival rate of mice was monitored for 10 days. According to the infection survival rate test, oral infection with bacteria led to 50% death at day 5 after infection. At day 7 postinfection, none of the mice in the wild-type SL1344 control group survived. In the fraxetin-treated control group, on day 5 after infection challenge, the mortality rate was similar to that in the infected group. At day 7 after infection, the mortality rate decreased to 60%. The blank control group did not die during the survival assessment (Fig. 5D). Based on these data, fraxetin improves survival outcomes in an *in vivo* model of *S.* Typhimurium infection.

## DISCUSSION

Fraxetin is a single compound of traditional Chinese herbs with various functions and is extracted from the traditional medicinal plant *Fraxinus* spp. Previous studies have reported that fraxetin has antibacterial activities against S. aureus (21). However, the role of fraxetin in *S.* Typhimurium infection has not been characterized. This study found that fraxetin inhibits *S.* Typhimurium invasion by inhibiting the transcription of genes in a feed-forward regulatory loop. In addition, these findings provide a mechanistic justification for the application of fraxetin in the treatment of *S.* Typhimurium infection. Fraxetin has multiple bioactivities, including scavenging reactive oxygen species and having hypoglycemic, antiosteoporosis, and antiplatelet activities (13–20, 23–25). For example, fraxetin can serve as a therapeutic agent for myocardial infarction (26) and protect rat brains from cerebral stroke by activating PI3K/Akt pathway and promoting angiogenesis (27). Fraxetin can partially protect against rotenone toxicity affecting the main protection system of the cells against oxidative injury (28). Our data showed firstly that fraxetin is a potential agent targeting the *S.* Typhimurium T3SS and can protect mice against *Salmonella.* However, we cannot say that the protection for *Salmonella*-infected mice is entirely due to the antivirulence of fraxetin. This is a complex process, and we speculate that the antioxidant and anti-inflammatory activity of fraxetin may also be involved in this protective process.

In this study, fraxetin was first proven to be an inhibitor of the *S.* Typhimurium T3SS. The increasingly serious problem of antibiotic resistance has limited the clinical application of antibiotics (29). Antibiotics exert antibacterial effects by inhibiting a major ingredient for bacterial growth, such as cell wall synthesis (e.g., penicillins), protein synthesis (e.g., macrolides), and DNA replication (e.g., quinolones). Therefore, it is important to explore new antibacterial infection strategies to solve the current problem of drug resistance. Fraxetin exerts an antivirulence effect by targeting the T3SS of *S.* Typhimurium instead of inhibiting the essential components of bacterial growth and is a promising complementary therapeutic drug for the clinical application of antibiotics.

There are several strengths to the application of fraxetin in clinical treatment. Fraxetin is widely available, has a low cost of preparation, and has been proven to have few side effects in clinical treatment (21, 30). The emergence of multidrug-resistant *S.* Typhimurium is now a global public health emergency (3). The fraxetin anti-*S.* Typhimurium infection mechanism is complex *in vivo*. Previous research reports have proven that fraxetin is able to suppress the oxidative damage by augmenting the endogenous antioxidant system and thus ameliorating the plasmodium infection in mice (31). It is likely that fraxetin can improve the survival rate of mice by inhibiting the activity of T3SS and effectively protecting the body from oxidative stress damage caused by *Salmonella* (32).

This study explored the mechanism for the inhibitory effects of fraxetin on *S.* Typhimurium T3SS. However, the regulatory mechanism of fraxetin on the T3SS transcriptional regulator AraC/XylS family remains to be clarified. In addition, fraxetin effectively protected mice from *S.* Typhimurium infection. However, the plasma concentration and half-life of fraxetin in fraxetin-treated mice infected with *S.* Typhimurium are unknown. Thus, the deeper regulatory mechanism of drugs for the AraC/XylS family and the pharmacokinetic parameters of fraxetin in *Salmonella*-infected mice still need to be further investigated in the future. Collectively, these results demonstrate that fraxetin inhibits *S.* Typhimurium infection by targeting the T3SS and may serve as a potential agent for the treatment of *S.* Typhimurium infection.

## MATERIALS AND METHODS

### Bacterial strains and culture conditions.

The *S.* Typhimurium strains and cell lines used during this study are listed in Table 1. All cultures were grown aerobically in Luria-Bertani (LB) broth with the addition of suitable antibiotics at 37°C. To induce SPI-1 gene expression, the cultures were subcultured in LB broth supplemented with 0.3 M NaCl.

**TABLE 1:** Cell line and bacterial strains used in this study

Cell line or bacterial strain	Description	Source
Cell line		
HeLa	Cervical carcinoma	ATCC
*S*. Typhimurium strain		
SL1344 WT	30 μg/mL streptomycin	Xiaoyun Liu, Peking University, China
SL1344 Δ*invA*	30 μg/mL streptomycin	Xiaoyun Liu, Peking University, China
SL1344 SipA-beta-lactamase (SipA-TEM)	SL1344 carrying the vector of SipA-TEM (100 μg/mL ampicillin)	This study
SL1344 Δ*invA* SipA-TEM	SL1344 Δ*invA* carrying the vector of SipA-TEM (100 μg/mL ampicillin)	This study
SL1344 SipB-3×Flag (SipB-3×Flag)	SL1344 chromosomally expressing Flag-tagged SipB (30 μg/mL streptomycin)	This study

### Determination of the MIC.

The MIC of fraxetin for *S*. Typhimurium SL1344 was determined using the broth microdilution method according to the Clinical and Laboratory Standards Institute (33). Overnight cultures were diluted with LB broth to an optical density (OD) of 0.1 at 600 nm (OD_600_), and 100 μL of the diluted culture was added to a 96-well plate. Each well was diluted by serial 2-fold dilutions (2,048 to 2 μg/mL) of fraxetin (purity > 98%; CAS number 574-84-5; Heibpurify, Chengdu, China). Twenty-four hours later, the lowest concentration of fraxetin that had no visible bacterial growth was defined as the MIC of fraxetin for SL1344.

### Swimming motility assay.

The swimming motility assay was performed as previously described (34). An approximate volume (10 mL) of LB medium (0.3% [wt/vol] agar) was added to different concentrations of fraxetin (0, 4, 8, 16, and 32 μg/mL) and plated into 10-cm plates. The negative control group was treated with the same volume of dimethyl sulfoxide (DMSO) with fraxetin. Each well of semisolid medium was inoculated with 5 μL of overnight culture (OD_600_ = 0.5) of SL1344 and incubated at 37°C for 7 h. The diameter (mm) of SL1344 colonies in different groups (each group had 3 replicates) was measured to assess swimming motility.

### *In vitro* growth curve.

Overnight cultures of SL1344 were diluted with 0.3 M NaCl LB to an OD_600_ of 0.1. Then, gradient concentrations of fraxetin were added to the bacterial suspensions. The suspensions were incubated at 37°C for 8 h. The growth curve was determined by measuring the OD_600_ at an interval of 1 h.

### Cytotoxicity assay.

To examine the fraxetin cytotoxicity to HeLa cells, HeLa cells (1 × 10^4^/well) were seeded into 96-well plates and incubated for 24 h. The cells were washed three times and then treated with new medium containing gradient concentrations of fraxetin for 12 h at 37°C. Lactate dehydrogenase (LDH) release was detected by an LDH detection kit (11644793001; Roche) according to the manufacturer's protocol. DMSO served as a mock treatment.

### Invasion assay.

The experimental protocol of bacterial invasion of HeLa cells was adapted from the gentamicin protection assay described previously (35). Briefly, *S.* Typhimurium was grown overnight and then subcultured (1:30) in different concentrations (0, 8, 16, and 32 μg/mL) of fraxetin for 4 h. HeLa cells (2 × 10^5^/well) were cultured in 24-well plates, washed three times with phosphate-buffered saline (PBS), and incubated with 500 μL of Dulbecco’s modified Eagle’s medium (DMEM) containing *S.* Typhimurium culture samples (MOI = 20) for another 2 h. Cells were washed three times with PBS and then treated with gentamicin (100 μg/mL) for 30 min before being lysed in 0.02% (vol/vol) Triton X-100 to determine the CFU of intracellular bacteria. For fluorescence microscopy, HeLa cells were seeded onto coverslips and infected as described above. Infected cells were fixed with 4% paraformaldehyde (20 min at room temperature [RT]) and then permeabilized with 0.02% (vol/vol) Triton X-100 (5 min, RT). Fixed cells were blocked with 4% goat serum (20 min, RT). *Salmonella* was labeled with anti-*S.* Typhimurium antibody (1 h, RT) (ab35156; Abcam), and the coverslips were exposed to Texas Red goat anti-rabbit IgG H&L (T2767; Life Technologies) for 30 min. Finally, Hoechst 33342 (C1025; Beyotime, China) was used for nuclear staining for 5 min. Images were visualized using an Olympus IX83 fluorescence microscope.

### The β-lactamase assay.

The β-lactamase (TEM) assay was carried out using a SipA-TEM fusion as a translocation reporter with some modifications (36). HeLa cells were plated in 96-well plates at 1.2 × 10^4^ cells per well. The overnight cultured SL1344 wild-type strain or Δ*invA* strain expressing SipA-TEM was inoculated into 0.3 M NaCl LB medium at a ratio of 1/30, followed by the addition of fraxetin (0, 8, 16, and 32 μg/mL) and incubation for 4 h at 37°C. The HeLa cells were infected with the SL1344 cultures (MOI = 20) for 2 h. The cells were washed twice with Hanks’ balanced salt solution (HBSS) to remove uninfected bacteria, and 120 μL of HBSS containing 20 μL of 6× CCF4/AM reaction mixture (K1095; Thermo Fisher) was then added. Following incubation at RT for 1 h, the hydrolysis of CCF4/AM was observed by fluorescence microscopy. CCF4/AM was used as an indicator of translocation. The CCF4/AM was blue when CCF4/AM was added to the cells and hydrolyzed by TEM, while the unhydrolyzed CCF4/AM was green.

### Secretion assay of T3SS effectors.

High salt stimulus can induce the secretion of T3SS effector proteins. To examine the effect of fraxetin on the secretion of T3SS effectors, SL1344 cells were cultured in LB broth with 0.3 M NaCl. Gradient concentrations of fraxetin (0, 8, 16, and 32 μg/mL) were then added to the cultures. Culture supernatants were collected by centrifugation at 14,000 × *g* for 20 min. The total proteins of 1.5 mL supernatant were precipitated with 10% trichloroacetic acid (TCA) (T0699; Sigma). The pellets were washed twice with acetone, resuspended in SDS-PAGE sample-loading buffer, analyzed by SDS-PAGE, and detected by staining with Coomassie blue.

### Immunoblotting.

To test the expression of SipA-TEM in the SL1344 SipA-TEM strain plasmid and SipB-3×flag genome expressed in SL1344 cells, overnight cultures were diluted with 0.3 M NaCl LB at a ratio of 1:30. Fraxetin was added to the cultures and incubated for 4 h at 37°C with agitation. The cultures were centrifuged for 5 min at 12,000 × *g*, and the pellets were resolved in 100 μL of SDS loading buffer and then analyzed by SDS-PAGE. Then, the transfer membranes were blocked with 5% skim milk for 1 h, followed by incubation with the appropriate primary antibodies, including rabbit anti-HilA IgG (1:500, prepared by our laboratory), rabbit anti-SipA IgG (1:1,000, prepared by our laboratory), rabbit anti-isocitrate dehydrogenase (ICDH) IgG (1:20,000; ABS2090; Sigma), and mouse anti-Flag IgG (1:5,000; F1804; Sigma). The rabbit HilA polyclonal antibody IgG and rabbit SipA polyclonal antibody IgG were prepared by our laboratory and purified according to a previously described protocol (37, 38). ICDH was used as a loading control. The membranes were washed three times and incubated with appropriate secondary antibodies (ab175775 and ab175781; Abcam), and the results were detected by an Odyssey CLx imaging system (Li-Cor).

### Quantitative real-time PCR.

The overnight cultured SipB-3×Flag strain was inoculated into 0.3 M NaCl LB medium. Gradient concentrations of fraxetin (0, 8, 16, and 32 μg/mL) were added to the cultures and incubated at 37°C for 4 h. Bacterial cells were collected by centrifugation at 12,000 × *g* for 10 min at 4°C. RNA extraction was performed using a bacterial total RNA extraction kit (B518625; Sangon Biotech) according to the manufacturer’s instructions. cDNA was obtained by using a RevertAid RT reverse transcription kit (K1691; Thermo Scientific). All qRT-PCR was carried out using SYBR green fluorescent dye (KTSM1401; AlpaLife). The sequences of the primers are listed in Table 2. As the reference gene, DNA gyrase subunit B (*gyrB*) was used to normalize gene expression (39). The relative levels of gene expression were determined according to the 2^-ΔΔCT^ method (40).

**TABLE 2:** Primers for RT-PCR in this study

Gene name	Primer sequence (5′–3′)	Product size (bp)
*sipA*	CCGGCACCTTGAAATGCAAA CGAATCCACACGCGAATGAC	385
*sipB*	ATGGGATGTATCGGGAAAGT CTCCATAATCGGGTTTAGCG	360
*sipC*	CAGCTTCGCAATCCGTTAGC TCAGCCTGGTTCAACGTCAG	358
*hilA*	TATCTCCGGGCAGATGATAC TCTGAGCAAAAGATTCGCAA	340
*hilC*	AGCGTATCAAGTCTGAAGCG ATCATAGCCACACATCGTCG	147
*hilD*	TAACGTGACGCTTGAAGAGG GGTACCGCCATTTTGGTTTG	123
*rtsA*	AGGTGGGGAGCATTGAAT CGTAATTGAAATTTTACCC	125
*gyrB*	TCATTTCCACTACGAAGGCG CCGAAAAAGACGGTATCGG	111

### Animal experiments.

All animal experiments were approved by the Institutional Animal Care and Use Committee of Jilin University (permit number 2021121809F). Female BALB/c mice (6 to 8 weeks old and 18 to 20 g) used for mouse infection were purchased from Liaoning Changsheng Biotechnology Co. Mice were randomly divided into the following three groups (*n* = 5 for each group): the SL1344 infection group, fraxetin treatment group, and PBS group. All groups of mice were fed water containing streptomycin (5 g/L) for 3 days. For the infection group, mice were orally infected with 5 × 10^6^ CFU of *S.* Typhimurium suspended in PBS using a gavage needle or 1 × 10^7^ CFU for survival assay. For the treatment group, the mice were given fraxetin by oral administration (100 mg/kg of body weight) and orally infected with 5 × 10^6^ CFU of *S.* Typhimurium 2 h post fraxetin administration. Fraxetin was offered at 12-h intervals for another 4 consecutive days. For the PBS group, mice were orally given the same volume of PBS as the bacterial suspension of the infected group. Mice were dissected 4 days postinfection, and tissue samples from the spleens and livers were homogenized in phosphate-buffered saline. Appropriate volumes (10 μL) of homogenate serial dilutions were dropped and cultured onto LB agar plates to determine the number of CFU. For survival assays, the survival rate of the mice was monitored for a consecutive 10 days.

### Ethics statement.

The animal study was reviewed and approved by The Institutional Animal Care and Use Committee of Jilin University (permit number 2021121809F).

### Statistical analysis.

The experimental data were analyzed by unpaired two-tailed *t* tests using GraphPad Prism 8.0 (GraphPad software, La Jolla, CA), except for the data from the mouse survival assay, which were assessed using the log rank test. *P* values are indicated in the figures as follows: ***, *P < *0.001; **, *P < *0.01; *, *P < *0.05. All experiments were performed in triplicate. All data are presented as the mean ± standard deviation.
